# SNX10 in autosomal recessive osteosclerosis, osteosarcoma, rheumatoid arthritis, and osteoporosis: molecular mechanisms and therapeutic implications

**DOI:** 10.3389/fcell.2025.1602240

**Published:** 2025-06-10

**Authors:** Sijia Liu, Huili Deng, Junjie Liu, Jun Zhang, Xier Chen, Xuchang Zhou, Chengqiang Zheng

**Affiliations:** ^1^ School of Sports and Health, Chengdu University of Traditional Chinese Medicine, Chengdu, China; ^2^ Department of Rehabilitation Medicine, The First Affiliated Hospital of Xiamen University, School of Medicine, Xiamen University, Xiamen, China; ^3^ School of Basic Medicine, China Three Gorges University, Yichang, China

**Keywords:** SNX10, autosomal recessive osteosclerosis, osteosarcoma, rheumatoid arthritis, osteoporosis

## Abstract

Bone metabolic diseases are typically caused by abnormal cell metabolism and cell death within the bone, involving cell types such as osteoblasts, osteoclasts, osteocytes, chondrocytes, and bone marrow mesenchymal stem cells. Bone metabolic diseases include autosomal recessive^osteosclerosis^ (ARO), osteosarcoma (OS), rheumatoid arthritis (RA), and osteoporosis (OP). However, there are other categories of bone metabolic disorders in addition to the four mentioned in this review, including, but not limited to, osteochondrosis, Paget’s disease, and hyperparathyroidism-associated bone disease, and others. The incidence of bone metabolism-related diseases has gradually increased over time and social changes, affecting a wider and wider group of people. Therefore, systematically analyzing the molecular pathological mechanisms of bone metabolic diseases, particularly the spatiotemporal dynamics of key regulatory nodes, has become an urgent need for developing novel therapeutic strategies. It is important to note that strictly speaking OS and RA are not usually categorized as bone metabolic disorders. However, this review categorizes them as bone metabolic diseases because of the pathological mechanisms, cellular metabolic abnormalities, and clinical evidence explored in OS and RA. Both OS and RA fit the basic profile of bone metabolic diseases. SNX10, as a member of the sorting nexin family, exerts unique regulatory functions in membrane transport through its phospholipid-binding properties mediated by the PX (phox homology) domain. Recent mechanistic analyses have shown that SNX10 exhibits multidimensional therapeutic potential in bone metabolic diseases by regulating pathways such as vesicle transport, lysosome maturation, and RANKL signal transduction. This review systematically integrates the latest research evidence on SNX10 in bone metabolic diseases, focusing on elucidating its molecular regulatory networks in conditions such as ARO, OS, RA, and OP, aiming to provide a theoretical basis for the application of SNX10-targeted precision therapeutic strategies in bone metabolic diseases.

## 1 Introduction

Bone metabolic diseases encompass a wide range of disorders, including but not limited to osteomalacia, Paget’s disease, hyperparathyroidism-related bone disease, and others. However, this review focuses on four bone metabolism-related diseases, namely, ARO, OS, RA and OP. Bone homeostasis essentially relies on the precise coupling between osteoblast-mediated bone formation and osteoclast-driven bone resorption (Lerner et al., 2019). Imbalance in the bone formation-resorption coupling can lead to various pathological phenotypes, including ARO, OS, RA, and OP. Autosomal recessive ossification is caused by impaired osteoclast activity (Udupa et al., 2023), while OS arises from uncontrolled osteoblastic differentiation of mesenchymal stem cells (Velletri et al., 2016). RA involves periarticular and subchondral bone erosion due to osteoclast differentiation and activation (Panagopoulos and Lambrou, 2018), whereas OP is characterized by an imbalance between bone resorption and formation. Therefore, a deep understanding of cellular activities and their underlying regulatory mechanisms in bone metabolism is crucial for the prevention and treatment of bone metabolic diseases.

The skeleton, as a mechanosensitive organ, undergoes lifelong modeling and remodeling in response to various physiological and pathological stimuli, particularly mechanical stress (Liang et al., 2021). During physiological bone remodeling, osteoclast-mediated bone resorption and osteoblast-driven bone formation are dynamically coupled through RANKL/OPG signaling molecules secreted by osteocytes. Together, these components form the “basic multicellular unit” (BMU) that maintains bone homeostasis (Jilka, 2003). Regulating the coupling of bone formation and resorption is a fundamental strategy for the prevention and treatment of bone metabolic diseases. Bone marrow mesenchymal stem/stromal cells (BMSCs), as a reservoir of osteoprogenitor cells, regulate their differentiation into the osteoblastic lineage through the Wnt/β-catenin and BMP/Smad pathways (Zhu et al., 2023). During the initiation of bone remodeling, osteocytes respond to mechanical stress by triggering the recruitment of osteoclast precursors. Mature osteoclasts acidify the microenvironment to dissolve the mineralized matrix, releasing growth factors like TGF-β and IGF-1, which create a chemotactic gradient that guides BMSCs to migrate toward the resorption lacunae. BMSCs differentiate into functional osteoblasts through the Runx2/Osterix transcriptional program, completing bone defect repair. BMSCs are pluripotent stem cells with self-renewal, immunomodulation, and multidirectional differentiation potential, with the advantages of easy access, low immunogenicity, and secretion of trophic factors (Zhou et al., 2022). BMSCs have been reported to replace and repair damaged tissues directly through cell proliferation and differentiation and indirectly through paracrine (Shi et al., 2010). Therefore, cell therapy based on BMSCs shows great potential in the treatment of bone metabolic diseases (Zhou et al., 2022).

Sorting nexin 10 (SNX10) is part of the sorting nexin family, which consists of 33 members. This family maintains intracellular membrane system homeostasis by regulating endosomal sorting, membrane protein recycling, and endosome-to-Golgi transport (Ye et al., 2015; Barnea-Zohar et al., 2021). All members of this family possess a characteristic phox homology (PX) domain, whose sequence diversity imparts specific functions to different SNX proteins in clathrin-dependent and independent endocytic pathways (Ye et al., 2015; Cullen, 2008; Zhou et al., 2016; Zhu et al., 2012; Worby and Dixon, 2002; Teasdale and Collins, 2012). The PX domain achieves subcellular localization by specifically recognizing phosphatidylinositol lipids, such as PI3P. This drives SNX proteins to target membranous compartments like early endosomes and recycling endosomes, thereby regulating the assembly of membrane transport complexes (Cullen, 2008). The core role of the PX domain in the yeast vacuolar protein sorting (VPS) system suggests that SNX family members have a universal regulatory function in intracellular membrane transport and cargo sorting in eukaryotes (Zhu et al., 2012). However, SNX10 is the only member of the family with vesicular-type ATPase (V-ATPase) trafficking, and a study confirmed that SNX10 mutations are directly associated with the development of ARO (17). SNX10 localizes to early endosomes in osteoclasts by binding to phosphatidylinositol 3-phosphate (PI3P). It regulates the nuclear translocation of NFATc1 downstream of the RANKL signaling pathway, playing a crucial role in the coupling of bone resorption and formation (Udupa et al., 2023; Battaglino et al., 2019). This review systematically integrates the multidimensional regulatory network of SNX10 in bone metabolic diseases, focusing on its molecular mechanisms affecting bone homeostasis through V-ATPase trafficking, RANKL signal transduction, and endosome maturation regulation. It provides a theoretical framework for developing targeted therapeutic strategies against SNX10.

## 2 Overview of SNX10

**FIGURE 1:**
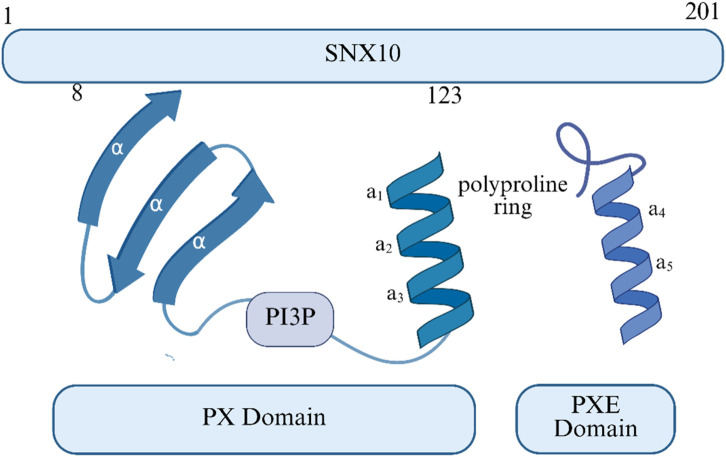
The SNX10 structure or structural domain.

SNX10 consists of an N-terminal PX domain and a C-terminal region rich in serine residues and negatively charged amino acids (Stein et al., 2020) (as shown in Figure 1). It plays a key role in cargo sorting within the endosomal pathway by binding to PI3P in early endosomes (Xu et al., 2013). The PX domain extends from exons 3 to 6 (c.30-381) and further extends into a PXe domain at the C-terminus (Xu et al., 2013; Xu et al., 2014). The structures reveal that SNX11 contains a novel PX domain, hereby named the extended PX (PXe) domain, with two additional α-helices at the C terminus. We demonstrate that these α-helices are indispensible for the *in vitro* functions of SNX11. We propose that this PXe domain is present in SNX10 and is responsible for the vacuolation activity of SNX10 (17). Studies suggest that the Arg16Leu mutation may affect SNX10’s binding with interaction partners without impacting its structural integrity. In contrast, Tyr32 and Arg51 are crucial for maintaining structural stability, and their mutations may affect binding with PI3P. SNX10 primarily localizes to the endosomes in mammalian cells (Qin et al., 2006). Its loss-of-function mutations can prevent the V-ATPase complex from targeting transport vesicles to the ruffled border, leading to impaired acidification of the resorption lacunae (Udupa et al., 2023; Xu et al., 2021). This molecular cascade defect ultimately results in the inactivation of osteoclast bone resorption function. In RANKL-induced osteoclast differentiation models, SNX10 is upregulated and is highly expressed during mouse embryonic bone development, further confirming its role in mediating V-ATPase transport to the ruffled border for acidification of the bone resorption microenvironment. In summary, SNX10 plays a crucial role in osteoclast bone resorption by regulating the subcellular localization of V-ATPase.

## 3 The role of SNX10 in autosomal recessive osteosclerosis

ARO is a rare, life-threatening genetic disorder and the most common form of osteodysplasia. It is characterized by increased bone density due to reduced osteoclast activity, and the presence of non-functional osteoclasts. The estimated incidence is 1 in 250,000 newborns (Palagano et al., 2018). ARO can be classified into an infantile (or “malignant”) form and a milder intermediate form (IARO) (Sobacchi et al., 2013; Askmyr et al., 2008). In malignant ARO, skeletal defects are present before birth, and nerve damage due to compression is irreversible (Stattin et al., 2017). ARO is usually fatal if not treated promptly with hematopoietic stem cell transplantation (HSCT) to produce functional osteoclasts.

ARO exhibits genetic heterogeneity, involving at least eight related genes: *TCIRG1, CLCN7, SNX10*, *OSTM1, PLEKHM1, CAII, TNFSF11/RANKL*, and *TNFRSF11A/RANK* (as shown in Table 1). The genes *TCIRG1, CLCN7, PLEKHM1, OSTM1, CAII*, and *SNX10* provide effector functions for osteoclasts, while *RANK* and *RANKL* are involved in osteoclast formation and differentiation (Koçak et al., 2019; Guerrini et al., 2008; Van Wesenbeeck et al., 2007; Sobacchi et al., 2007; Chalhoub et al., 2003; Kornak et al., 2001; Frattini et al., 2000; Sly et al., 1983). *PLEKHM1* and *SNX10* mutations affect vesicular transport within osteoclasts, causing a lack of ruffled borders. Generally, ARO due to *PLEKHM1* or *SNX10* gene mutations can be diagnosed at an early stage. Defects in osteoclast differentiation and function may be a potential pathogenic mechanism for ARO. Therefore, hematopoietic stem cell transplantation (HSCT) holds the potential to rebuild a healthy hematopoietic system, thereby repairing or replacing dysfunctional osteoclasts to improve the condition. For severe patients, HSCT can be a potential therapeutic option (Steward, 2010). However, the feasibility of HSCT must be comprehensively evaluated based on ARO patients’ specific conditions, transplantation risks, and prognosis (Palagano et al., 2018; Sobacchi et al., 2013).

**TABLE 1:** Eight genetic mechanisms in ARO pathogenesis.

Authors	Genes	Pathogenesis	The function of genes
Van Wesenbeeck et al. (2007)	*PLEKHM1*	gene mutation	Regulates autophagy-lysosome fusion and bone resorption
Sly et al. (1983)	*CAII*	gene mutation	Regulates osteoclast function via proton secretion in bone resorption
Capo et al. (2022)	*TCIRG1*	gene mutation	Essential for osteoclast proton pump in bone resorption
Schrecker et al. (2020)	*CLCN7*	gene mutation	Critical for osteoclast acidification during bone resorption
Huybrechts and Van Hul (2022)	*SNX10*	gene mutation	Regulates osteoclast vesicular trafficking for bone resorption
Vacher et al. (2020)	*OSTM1*	gene mutation	Essential for osteoclast lysosomal function and bone resorption
Kong et al. (1999)	*TNFSF11/RANKL*	gene mutation	Essential ligand for osteoclast differentiation and activation
Kong et al. (1999)	*TNFRSF11A/RANK*	gene mutation	Essential receptor for osteoclastogenesis and bone remodeling

The large deletion in the 5′untranslated region (UTR) of *SNX10* leads to the loss of its transcripts, resulting in abnormal osteoclast activity (Udupa et al., 2023). Studies have shown that SNX10 overexpression can induce the accumulation of large vacuoles on the cell membrane, potentially mediated through its interaction with V-ATPase. Given the critical role of V-ATPase in osteoclasts and its binding to SNX10 via the v1d subunit, it is hypothesized that SNX10 may play a significant role in targeting V-ATPase to the ruffled border (Xu et al., 2014). Notably, SNX10-induced vacuole formation depends on V-ATPase function, while the Arg16Leu mutant might lie outside the V-ATPase interaction domain. However, other proteins interacting with SNX10 could be crucial for V-ATPase transport, and mutations might affect these interactions, leading to osteoclast dysfunction (Xu et al., 2014). Additionally, SNX10 deficiency inhibits endocytosis, severely impairing ruffled border formation and blocking bone resorption activity, suggesting SNX10 mediates bone resorption and acidification through vesicle transport regulation (Battaglino et al., 2019). The R51Q mutation in SNX10 has been confirmed as a pathogenic factor in human ARO. In the first R51Q SNX10 knock-in mouse model, osteocyte necrosis resulted from impaired osteoclast activity, with mutant osteoclasts lacking ruffled borders and proton secretion functions. These findings confirm the R51Q mutation in SNX10 as a causative factor for ARO and provide an important model system for studying this rare disease (Stein et al., 2020).

Using *RANKL*-stimulated peripheral blood monocytes, we found that osteoclasts could be generated from cells isolated from patients carrying the SNX10 splice site mutation. This finding is consistent with previous studies, which successfully generated osteoclasts from peripheral blood of patients with other SNX10 mutations, as well as from spleen or bone marrow cells of Snx10 knockout (KO) mice. In contrast, silencing Snx10 in the mouse pre-osteoclastic cell line Raw 264.7 was shown to inhibit RANKL-induced osteoclast differentiation (Ye et al., 2015; Zhou et al., 2016). Moreover, SNX10 can inhibit *RANKL*-induced osteoclast differentiation. During *RANKL*-stimulated osteoclastogenesis, *SNX10* mRNA levels increase significantly, aligning with its role in osteoclast function rather than formation (Zhu et al., 2012; Stattin et al., 2017). Given the high incidence of ARO in regions with consanguinity, SNX10 might be associated with “Westenberg’s osteosclerosis” and other ARO cases with unclear molecular mechanisms (Pangrazio et al., 2013).

Clinical studies show that the severity of SNX10-dependent ARO exhibits significant heterogeneity and is not associated with specific molecular defects. All patients display prominent symptoms in infancy, allowing for early diagnosis, and present with anemia at diagnosis or in their medical history, but without significant immunodeficiency. Notably, most patients experience secondary neurological defects with varying symptoms. These patients received hematopoietic stem cell transplantation at different stages. Overall, the clinical outcomes for SNX10-dependent ARO are more favorable compared to those dependent on *TCIRG1* (Pangrazio et al., 2013).

Studies have shown that Tyr32 and Arg51 are essential for SNX10 protein stability and vacuolization activity, whereas the Arg16Leu mutation may interfere with SNX10 function in osteoblasts by affecting protein interactions (Xu et al., 2014). Additionally, about 50% of ARO patients carry mutations in the *TCIRG1* gene, which encodes the osteoclast-specific a3 subunit of V-ATPase, essential for resorption lacuna acidification and vesicle transport. Various mutation types exist in the *TCIRG1* gene, including missense mutations, nonsense mutations, small deletions/insertions, splice site mutations, and large deletions (Rabi, 2019). Loss-of-function mutations in *TCIRG1* lead to failures in resorption lacuna acidification, affecting bone resorption. Recent studies suggest that the human *SNX10* gene is a novel target in osteonecrosis formation, with its encoded product potentially being a key regulatory factor for V-ATPase budding or targeting at the ruffled border. Mutations in the *SNX10* gene may result in “secondary V-ATPase deficiency”, further impacting osteoclast function (Koçak et al., 2019).

**FIGURE 2:**
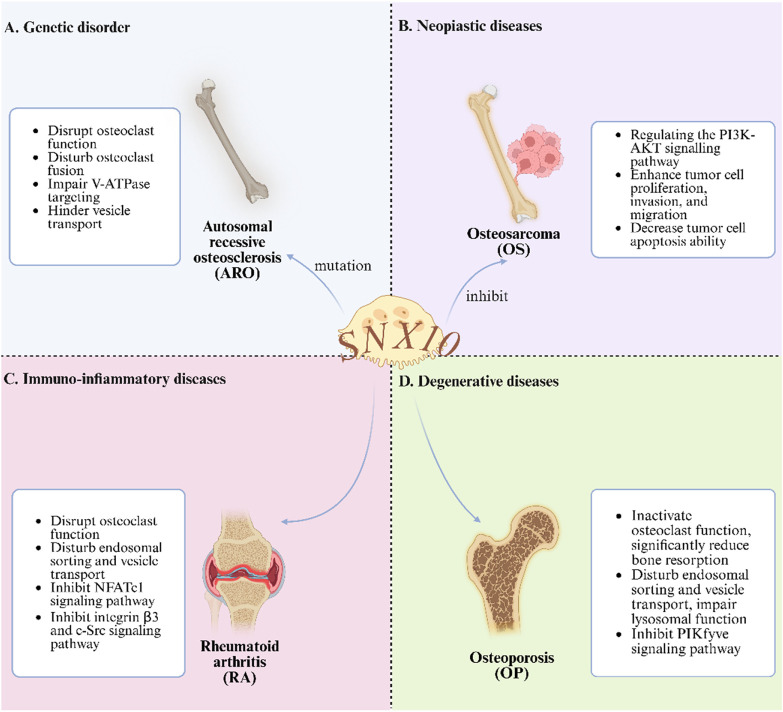
The Role of SNX10 in Bone Metabolic Diseases. **(A)** Interfering with osteoclast function etc., in genetic disorder; **(B)** Regulating signaling pathways to inhibit tumors in neoplastic diseases; **(C)** Disrupting osteoclast - related physiological processes in immuno - inflammatory diseases; **(D)** inactivating endosomal function to affect bone resorption in degenerative diseases).

In summary, ARO primarily occurs in regions with high rates of consanguinity. Although its incidence is low, it poses a potential threat to life. The pathogenic mechanisms are complex and varied, including large deletions in the 5′untranslated region (UTR) of the *SNX10* gene, leading to the loss of SNX10 transcripts and abnormal osteoclast activity. Additionally, *RANKL* influences SNX10 function by stimulating osteoclastogenesis and inducing vacuole formation, contributing to the disease’s onset and progression. Notably, the R51Q mutation has been confirmed as one of the causative factors of ARO. Besides abnormalities in the *SNX10* gene, deletions in other untranslated regions and mutations in genes such as *TCIRG1, CLCN7, PLEKHM1, OSTM1*, and *CAII* may also lead to ARO. Future research should delve deeper into these genes and their regulatory networks to fully elucidate the molecular mechanisms of ARO, thereby providing a theoretical basis for developing effective treatments (As shown in the Figure 2A).

## 4 The role of SNX10 in osteosarcoma

OS is the most common malignant primary bone tumor in children and adolescents, accounting for approximately 20% of primary bone tumors (Mirabello et al., 2009). OS is characterized by a rapid progression as well as the lack of specificity of early clinical symptoms (Cai et al., 2021; Scott et al., 2018). The development of OS is closely related to the dysregulation of osteoclast and osteoblast production, which is a key factor in abnormal bone formation (Wang et al., 2023; Barnea-Zohar et al., 2024). Long non-coding RNAs (lncRNAs) are RNA molecules longer than 200 nucleotides (Mirabello et al., 2009). Studies have shown that lncRNAs regulate gene expression through interactions with proteins, miRNAs, and RNA-binding proteins, thereby playing critical roles in both physiological and pathological processes (Barnea-Zohar et al., 2021). In various tumors, abnormal lncRNA expression is closely associated with metastasis, drug resistance, and disease progression (Cai et al., 2021; Wang et al., 2023; Barnea-Zohar et al., 2024). MicroRNAs (miRNAs) are endogenous short RNAs, approximately 22 nucleotides in length, that regulate gene expression at the post-transcriptional level through base pairing (Vacher et al., 2020; Han and Liu, 2018). MiRNAs such as miR-301b significantly promote tumor cell proliferation, invasion, and migration by regulating target genes like *SNX10* in OS(37). Additionally, miR-21 limits OS cell migration and invasion by inhibiting the activity of matrix metalloproteinases (MMPs) (Schrecker et al., 2020; Vacher et al., 2020). These studies indicate that miRNAs play a crucial role in the occurrence and progression of OS.

LncRNAs and miRNAs interact to influence the progression of OS through cross-regulation. Previous studies have found that *SNHG12* significantly inhibits OS cell proliferation and regulates the cell cycle by modulating the miR-195-5p*/Notch2* axis (Scott et al., 2018). Additionally, LINC01614 may play a crucial role in OS function and development by indirectly regulating SNX3 and affecting the Wnt signaling pathway (Cai et al., 2021). These findings suggest that lncRNA-miRNA interactions play an important regulatory role in the pathology of OS, providing new insights into its molecular mechanisms. The regulatory roles of lncRNAs and miRNAs in OS offer a theoretical basis for their use as diagnostic markers and therapeutic targets (Yang et al., 2021). For instance, differentially expressed lncRNAs (such as *MALAT1* and *SNHG12*) and miRNAs (such as miR-301b and miR-21) are closely associated with the malignant progression of OS and can serve as potential prognostic markers (Diener et al., 2022). Future research should explore the specific regulatory mechanisms of lncRNAs and miRNAs in OS, particularly their roles in the tumor microenvironment. Combining high-throughput sequencing technologies with bioinformatics analysis can more comprehensively reveal the functional lncRNA-miRNA regulatory networks in OS (Sheng et al., 2023). Additionally, developing targeted therapeutic strategies based on lncRNAs and miRNAs holds promise for providing more effective treatment options for OS patients.

Dual-luciferase reporter assays have further revealed a functional antagonistic relationship between miR-301b and SNX10 (46). Experimental results indicate that miR-301b specifically binds to the 3′ UTR region of SNX10 mRNA, thereby inhibiting its expression. In HEK293T cells, luciferase activity was significantly decreased after transfection with miR-301b mimics. Study exploring the role of miR-301b in OS, transfection conditions and controls were utilized. Transient transfection was carried out using Lipofectamine 2000 following the manufacturer’s instructions. Incubation times were specified in some experiments, such as 48 h for luciferase reporter assay and measurements at 0, 24, 48, and 72 h in the cell proliferation assay. However, the plasmid concentration was not mentioned. Regarding controls, a pcDNA3.1-empty vector was used to compare with pcDNA3.1-SNX10 transfection. A mutant (MUT) SNX10 was constructed and co-transfected with miR-301b mimics, along with wild-type (WT) SNX10, to verify the targeting relationship. Additionally, negative controls (NC) for miR-301b mimic/inhibitor were included to rule out non-specific effects. However, no similar change was observed in the MUT SNX10 group (SNX10 3′UTR mutant group), confirming the direct regulatory relationship between miR-301b and SNX10. Despite some important findings from the study, this exploration still has some minor or potential shortcomings: it only provides a preliminary characterization of the effects of miR-301b on OS through potential binding to SNX10 based on *in vitro* experiments, but ignores their precise binding effects and further molecular mechanisms. It is understood that the role of miR-301b in OS requires clinical patient data and detailed studies of specific pathways (Wang et al., 2023). Moreover, miR-301b significantly promotes the malignant progression of OS by inhibiting the expression of SNX10 and epithelial-mesenchymal transition (EMT)-related molecules (Wang et al., 2023). Low expression of miR-301b can significantly inhibit OS cell proliferation, invasion, and migration, while its overexpression markedly enhances these malignant phenotypes. Notably, upregulation of SNX10 can significantly reverse the oncogenic effects of miR-301b, highlighting the potential therapeutic value of miR-301b inhibitors in suppressing OS cell proliferation, migration, and invasion. Additionally, downregulation of miR-301b leads to a significant decrease in the expression levels of EMT-related markers, including N-cadherin, vimentin, and MMP9, further confirming the critical role of the miR-301b/SNX10 axis in OS metastasis (Barnea-Zohar et al., 2024).

In summary, early clinical symptoms of OS are often subtle. However, once metastasis occurs, the disease can rapidly progress to a life-threatening malignant phenotype. This study systematically reviews the regulatory mechanisms of miRNAs and lncRNAs in OS. MiR-34a significantly inhibits OS cell proliferation and invasion by targeting the Notch signaling pathway, while miR-301b plays a crucial role in cell proliferation, invasion, and migration by regulating target genes such as *SNX10*. Additionally, lncRNAs play important roles in OS progression. Specifically, lncRNA-HOTAIR promotes OS cell proliferation, migration, and invasion by activating the Wnt/β-catenin signaling pathway. LncRNA-MALAT1 affects tumor cell apoptosis and metastasis by sponging miRNAs. And lncRNA-TUG1 mediates tumor cell proliferation and chemotherapy resistance through the PI3K/AKT signaling pathway. Notably, the regulatory mechanisms of lncRNAs and miRNAs differ significantly, making lncRNAs promising biomarkers for OS prognosis prediction and potential targets for resistance therapy. Future research should focus on the regulatory effects of SNX10 and SNX3 on other miRNAs and further elucidate the coding mechanisms and interaction networks of miRNAs and lncRNAs. These studies will help to fully uncover the pathogenesis of OS and provide theoretical support for developing precise therapeutic strategies (As shown in Figure 2B).

## 5 The role of SNX10 in rheumatoid arthritis

Periarticular and subchondral bone erosion in RA is primarily caused by abnormal differentiation and activation of osteoclasts (Schett and Gravallese, 2012). Bone destruction is a significant clinical feature in RA patients (Shim et al., 2018). The ideal strategy for treating RA involves inhibiting osteoclast activity while also suppressing immune inflammation (Hascoët et al., 2023; Deng et al., 2021).

Activating osteoclastogenesis is dependent on the binding of nuclear factor κB receptor activator (RANK) to its specific ligand, RANKL (56). Osteoclast differentiation and its function are mainly regulated by the calmodulin/NFATc1 signaling pathway, in which NFATc1 functions as a core molecular switch (Battaglino et al., 2019). c-Src, a non-receptor tyrosine kinase, is highly expressed in osteoclasts, and its knockout results in impaired ruffled border formation and defective bone resorption function (Zhou et al., 2016). c-Src participates in adhesion-induced sealing zone (SZ) formation by phosphorylating proline-rich tyrosine kinase 2 (PYK2), with the SZ being a critical structure for osteoclast bone resorption (Duong et al., 1998; Shyu et al., 2007). Within the resorption lacuna formed by SZ closure, the plasma membrane further differentiates into the ruffled border, facilitating bone matrix degradation through the secretion of protons (H^+^) and lytic enzymes such as cathepsin K (CtsK) and matrix metalloproteinase 9 (MMP9) (Shyu et al., 2007).

RANKL stimulation significantly upregulates SNX10 mRNA expression in osteoclasts. SNX10 knockout does not impede osteoclast differentiation but causes abnormalities in the actin ring structure and loss of sealing zone integrity (the core of which is composed of F-actin, surrounded by adhesion molecules such as integrins, vinculin, and paxillin). SNX10 deficiency significantly suppresses the expression of TRAP, CtsK, and MMP9, as well as bone resorption activity. Overexpression of SNX10 can partially reverse these phenotypes, confirming its direct role in regulating bone resorption through actin ring assembly (Battaglino et al., 2019; Sultana et al., 2020).

Osteoclasts achieve bone resorption through the ruffled border, a structure dependent on V-ATPase-mediated acidification. V-ATPase, a multi-subunit complex, is responsible for transporting protons produced by carbonic anhydrase II. The formation, structural stability, and functional maintenance of the SZ are significantly regulated by Src kinase activity. SNX10 plays a crucial role in pathological bone remodeling by regulating the endosomal sorting pathway. Studies have confirmed that SNX10 physically interacts with V-ATPase and regulates its subcellular localization (Aker et al., 2012). In summary, the regulation of V-ATPase by SNX10 may influence lysosomal acidification, indirectly modulating NFATc1 stability (Zhou et al., 2016), and providing a potential mechanistic explanation for SNX10-RANKL signaling interaction.

The dynamic reorganization of the osteoclast cytoskeleton strictly depends on the activation of αvβ3 integrin and its ligand-binding capability (Xiang et al., 2019). Studies have confirmed that αvβ3 integrin initiates the assembly of the SZ by mediating the polarized aggregation of F-actin precursor structures toward the bone matrix interface (Gramoun, 2010). Activated αvβ3 integrin induces actin stress fiber reorganization and membrane pseudopodia formation by activating Rho family GTPases (such as RhoA and Cdc42), thereby coordinately regulating osteoclast adhesion, migration, and bone resorption functions (Adams, 2002). In this process, the calmodulin (CaM)-dependent signaling pathway promotes NFATc1 dephosphorylation, facilitating its nuclear translocation and activation of downstream target genes (such as *TRAP, CtsK, MMP9*), ultimately driving osteoclast differentiation and osteolytic function. SNX10 knockout reduces NFATc1 protein stability through post-transcriptional regulatory mechanisms (not at the gene expression level). SNX10 deficiency causes V-ATPase to abnormally accumulate in endosomes/lysosomes, leading to excessive acidification and accelerating NFATc1 degradation via lysosomal protease pathways. The decrease in NFATc1 levels inhibits integrin β3 transcription through a negative feedback loop, reducing αvβ3 complex expression and ultimately blocking downstream c-Src/PYK2 signaling cascades (Zhou et al., 2016). In summary, SNX10 deficiency downregulates integrin β3 expression, inhibiting the activation of non-receptor tyrosine kinase c-Src and its downstream effector molecule PYK2(63), thereby disrupting the multimodal signaling network essential for osteoclast bone resorption function.

In the Paramecium model, inhibiting dynamin or Rab5-mediated endocytosis leads to the abnormal retention of EFF-1 fusion protein on the apical membrane, causing excessive cell fusion (Barnea-Zohar et al., 2021). This evolutionarily conserved membrane transport regulation mechanism suggests that SNX family proteins, through their PX domains, specifically target endosomal membranes to regulate sorting and transport pathways, which are crucial for maintaining tissue development homeostasis. SNX10-deficient osteoclasts exhibit abnormal fusion phenotypes directly related to SNX10 protein functional defects. The R51Q point mutation reduces SNX10 protein stability and alters domain conformation, disrupting its membrane transport regulation capability, and ultimately resulting in a complete loss of bone resorption function. Disruption of this regulatory mechanism leads to continuous fusion of mature osteoclasts without compensatory inhibitory pathways. In summary, the SNX10-R51Q mutant serves as a dominant-negative model, providing a theoretical basis for developing small molecule inhibitors targeting the SNX10-PX domain, which may selectively inhibit pathological bone resorption (Stein et al., 2020).

FK506 binding protein 12 (FKBP12), an immunophilin that regulates calcium signaling and protein interactions, modulates calmodulin (CaM) activity by promoting actin reorganization, ion channel regulation, and dephosphorylation of proteins related to endocytosis and vesicle transport (Battaglino et al., 2019). Yeast two-hybrid (Y2H) experiments show that SNX10 and FKBP12 have the highest confidence interaction in osteoclasts, suggesting a physiologically relevant functional synergy. FKBP12 regulates calcium ion homeostasis and channel gating by selectively binding to cellular receptors or target proteins (such as sarcoplasmic/endoplasmic reticulum calcium release channels), while its interaction with TGF-β type I receptor inhibits downstream signal transduction. Additionally, FKBP12 inhibits the signaling activity of the epidermal growth factor receptor (EGFR) by regulating its phosphorylation and coordinates CaM-mediated actin reorganization, ion channel function, and vesicle transport (Battaglino et al., 2019). SNX10 and FKBP12 co-immunopreciopitate in osteoclasts. Colocalization of Snx10 and FKBP12 was confirmed by immunohistochemistry in both multinucleated osteoclasts and gastric zymogenic cells. FKBP12 silencing inhibits osteoclast differentiation. The vesicular transport disruption caused by the loss of their functions can lead to the formation of abnormally large vacuoles, indicating their joint role in maintaining endosomal homeostasis and transport pathway integrity (Battaglino et al., 2019). In summary, the SNX10-FKBP12 complex may serve as a novel target for intervening in pathological bone resorption by dual regulation of calcium signaling and vesicle transport. Developing small molecule inhibitors to disrupt this interaction may offer new therapeutic strategies for bone metabolic diseases such as RA.

In summary, SNX10 plays a critical regulatory role in the pathological progression of RA by modulating the osteoclast functional axis. SNX10 is involved in endosomal sorting and co-immunoprecipitates with V-ATPase, regulating its intracellular transport. The absence of functional SNX10 results in severe dysfunction of osteoclasts, with significant impairments in endocytosis, extracellular acidification, ruffled border formation, and bone resorption. Knockout of SNX10 leads to abnormal actin bands and reduced podosome belts in osteoclasts, significantly inhibiting the production of TRAP, CtsK, and MMP9, as well as bone resorption activity, while overexpression of SNX10 can partially restore these activities. SNX10 knockout reduces NFATc1 protein expression in osteoclasts through transcription-independent mechanisms, accelerating its degradation. Given the critical role of osteoclasts in the pathogenesis of periarticular bone erosion in RA, the regulatory effect of SNX10 on osteoclasts makes it a potential therapeutic target for RA. The ideal RA treatment strategy involves inhibiting osteoclast activity while suppressing immune inflammation, and research on SNX10 provides a direction for achieving this strategy (As shown in Figure 2C).

## 6 The role of SNX10 in osteoporosis

The dynamic imbalance between bone resorption and formation is the core pathological mechanism of OP. A study indicates that approximately 4% of severe OS cases are caused by SNX10 mutations (Ye et al., 2015). However, this reference is temporally limited (published over a decade ago) and lacks corroborating contemporary studies, which may introduce potential biases in the stated conclusions. The *SNX10* gene plays a crucial regulatory role in the bone resorption function of osteoclasts (OCLs), primarily affecting cellular function regulation rather than the differentiation process (Battaglino et al., 2019; Sultana et al., 2020). The R51Q mutation in SNX10 results in the inactivation of osteoclast function. In wild-type mouse models, the fusion process of mononuclear precursor cells is precisely regulated, and the fusion capacity of mature osteoclasts is strictly limited. In contrast, mononuclear precursor cells carrying the homozygous R51Q SNX10 mutation undergo uncontrolled fusion, producing giant osteoclasts with functional defects, 10 to 100 times the size of wild-type cells. Notably, mutant osteoclasts exhibit significantly reduced endocytic activity, suggesting that SNX10 dysfunction may lead to dysregulated cell fusion by altering membrane system homeostasis (Barnea-Zohar et al., 2021). The R51Q SNX10 mutant protein exhibits decreased stability and altered lipid-binding properties, supporting the aforementioned mechanistic hypothesis and aligning with SNX10’s core function in vesicular transport (Barnea-Zohar et al., 2021). In summary, osteoclast volume and function are precisely controlled by an SNX10-dependent cell-autonomous regulatory mechanism, which maintains physiological homeostasis by negatively regulating the fusion of mature cells. The R51Q mutation disrupts this regulatory network, causing pathological cell hyperfusion and bone resorption dysfunction, ultimately leading to *in vivo* phenotypes of bone microstructure destruction (Barnea-Zohar et al., 2021).

Phosphatidylinositol 3-phosphate kinase (PI3P kinase, or PIKfyve) is essential for normal osteoclast function and lysosome formation. PIKfyve is a lipid kinase that targets endosomes through protein-lipid interactions between its Fyve domain and PI3P in the endosomal membrane (Dayam et al., 2017; Keule et al., 2016). It is reported that there are seven different types of membrane lipid phosphoinositides, which can interconvert through kinases and phosphatases. PIKfyve is one such kinase, as its product, PI (3,5)P2, has become a stress-induced signaling lipid and a key regulator of endolysosomal trafficking pathways, including endosomal sorting and membrane homeostasis (Keule et al., 2016; Rutherford et al., 2006). Apilimod, a nanomolar-specific PIKfyve inhibitor, can block the generation of the second messenger molecule PI (3,5)P_2_. It has been shown that the absence or inhibition of the PIKfyve enzyme leads to endosome enlargement and vacuolation, preventing the transition from endosomes to lysosomes (Qin et al., 2006; Hirano et al., 2015; de Lartigue et al., 2009). Studies show that in apilimod-resistant NHBL cell lines, the introduction of SNX10, OSTM1, and ClC7 can sensitize cells to the drug. Mutations in these three genes lead to severe OS in humans (Sultana et al., 2020). In clinical treatment of this disease, the tissue-specific effects of SNX10 mutations must be considered. Sole reliance on hematopoietic stem cell transplantation may fail to correct hypocalcemia and can sometimes result in fatal outcomes (Ye et al., 2015).

Previous studies have shown that PIKfyve and SNX10 colocalize in early endosomes and can co-precipitate from cell lysates, suggesting a direct interaction or joint participation in multiprotein complex assembly (Sultana et al., 2020). Lysosomes, as acidic membrane-bound organelles, receive degradable substrates through endocytosis, phagocytosis, and autophagy, relying on their enzymatic systems for catabolism. PIKfyve synthesizes lysosome-targeted phosphoinositide PI (3,5)P2 through its kinase activity, serving as a key regulatory factor in maintaining lysosomal homeostasis. Co-staining of endosomal and lysosomal markers in apilimod-treated osteoclasts derived from RAW264.7 cells shows significant endosomal enlargement and a marked reduction in lysosome numbers. This confirms that the loss of PIKfyve activity and reduced PI (3,5)P2 synthesis can lead to endosomal accumulation and inhibition of lysosome formation in osteoclasts (Sultana et al., 2020).

Inhibition of PIKfyve disrupts membrane trafficking pathways, leading to abnormal vesicle enlargement, impaired degradation capacity, ion homeostasis imbalance, and reduced autophagic flux, ultimately causing multifaceted physiological defects and significant inflammatory responses (Dayam et al., 2017; Choy et al., 2018). Inhibiting PIKfyve results in fewer but abnormally enlarged endolysosomes, indicating a disruption in the dynamic balance of fusion and fission, with fusion predominating (Choy et al., 2018). Gayle et al. confirm that the cytotoxic effects of apilimod in B-cell non-Hodgkin lymphoma depend on the expression of key osteoclast effector genes (*CLCN7, OSTM1, SNX10*) (Sultana et al., 2020). Moreover, SNX10 expression is required for drug response and may play a role by modulating drug-target affinity or maintaining PIKfyve residual activity. These findings collectively suggest that targeting PIKfyve and its inhibitor apilimod may regulate osteoclast differentiation and activity, offering new therapeutic targets for lysosome-related bone metabolic diseases such as OS (Sultana et al., 2020).

Comprehensive evidence indicates that SNX10 significantly reduces the pool of mature osteoclasts by negatively regulating the proliferation and differentiation cascade of osteoclast precursor cells. At the molecular level, SNX10 specifically inhibits the binding efficiency of the RANKL-RANK ligand-receptor by intervening in the dynamic balance of the RANKL/RANK/OPG signaling axis, thereby blocking critical signal transduction for osteoclast differentiation and maturation, and ultimately weakening their bone resorption capability. Notably, SNX10 continues to regulate the function of mature osteoclasts by reprogramming signaling networks such as MAPK/NF-κB and mitochondrial oxidative phosphorylation pathways, persistently inhibiting cellular resorptive activity. PIKfyve regulates the endolysosomal system by catalyzing the production of PI(3,5)P2, and its absence or inhibition results in endosomal enlargement and reduced lysosome formation. SNX10, OSTM1, and ClC7 are closely related to the function of *PIKFYVE*, and their mutations can lead to severe OS. Apilimod acts by inhibiting *PIKFYVE*, with SNX10 being a necessary condition for its drug response. Special clinical attention should be given to the tissue-specific effects of SNX10 mutations to prevent complications such as hypocalcemia. Further study of the molecular mechanisms of *PIKFYVE* and related proteins is essential for developing targeted therapies for OS (As shown in Figure 2D).

## 7 Conclusions and perspectives

The maintenance of skeletal system homeostasis relies on the coordinated actions of multiple cell types, including osteoblasts, osteoclasts, osteocytes, chondrocytes, and bone marrow mesenchymal stem cells (as shown in Figure 1). Abnormalities in these functional units are closely linked to the development of bone metabolic diseases. Disorders such as ARO, OS, RA, and OP are often driven by dysregulated cellular metabolism or aberrant activation of programmed cell death pathways. Previous studies have shown that SNX10 plays a critical regulatory role in bone metabolic diseases by modulating the proliferation, differentiation, and functional polarization of these cell populations. This review systematically examines the regulatory network of SNX10 in bone metabolic diseases, highlighting its significant therapeutic potential.

HSCT is the first-line treatment for ARO and can effectively reverse the osteosclerotic phenotype by reconstructing a functional osteoclast pool. Targeting downstream lncRNA molecules of SNX10, such as MAFG-AS1, can significantly inhibit the formation of pulmonary metastases in OS, offering new directions for RNA interference therapies. In RA, SNX10 shows potential therapeutic value by inhibiting osteoclast activity and modulating immune-inflammatory responses. Additionally, in OP, SNX10 may further inhibit osteoclast activity by regulating the PI3K/AKT and NF-κB signaling pathways, thereby reducing bone resorption and becoming a core therapeutic strategy. Future research should further explore the molecular mechanisms of SNX10 in various bone metabolic diseases to provide a theoretical basis for developing targeted therapeutic strategies.

Although SNX10 dysfunction is thought to directly contribute to the pathogenesis of bone metabolic diseases, its role as a causative factor in certain conditions requires further validation. For instance, in ARO, at least seven other genes (*TCIRG1, CLCN7, OSTM1, PLEKHM1, CAII, TNFSF11/RANKL,* and *TNFRSF11A/RANK*) are closely associated with its pathogenesis. We can investigate whether a specific gene, like *SNX10*, has the same regulatory effect on ARO. This complexity underscores the importance of clarifying the mechanisms to develop effective treatments. Investigating the direct regulation of OS in bone metabolic diseases by SNX10 will be the next direction of research, so that the regulation of bone metabolic diseases by SNX10 is not in conjunction with miRNAs. To study the exact mechanism of SNX10, including the complex biological processes and signaling pathways of OS development. We can also explore the mechanisms of other sorting nexin families in the regulation of bone metabolic diseases, such as SNX3 or SNX11 mentioned in the paper. In summary, therapeutic strategies targeting SNX10 abnormalities face numerous challenges that necessitate further in-depth research.
